# Effects of Variations in the Chemical Composition of Individual Rice Grains on the Eating Quality of Hybrid Indica Rice Based on Near-Infrared Spectroscopy

**DOI:** 10.3390/foods11172634

**Published:** 2022-08-30

**Authors:** Weimin Cheng, Zhuopin Xu, Shuang Fan, Pengfei Zhang, Jiafa Xia, Hui Wang, Yafeng Ye, Binmei Liu, Qi Wang, Yuejin Wu

**Affiliations:** 1 Hefei Institutes of Physical Science, Chinese Academy of Sciences, Hefei 230031, China; 2 Science Island Branch of Graduate School, University of Science and Technology of China, Hefei 230026, China; 3 Hainan Branch of the CAS Innovative Academy for Seed Design, Sanya 572025, China; 4 Rice Research Institute, Anhui Academy of Agricultural Sciences, Hefei 230041, China; 5 National Key Laboratory for New Variety Development of Hybrid Rice of Ministry of Agriculture, Anhui Win-All Hi-Tech Seed Co. Ltd., Hefei 230088, China

**Keywords:** near-infrared spectroscopy, hybrid indica rice, single-grain chemical composition, eating quality

## Abstract

The chemical composition of individual hybrid rice (F2) varieties varies owing to genetic differences between parental lines, and the effects of these differences on eating quality are unclear. In this study, based on a self-developed near-infrared spectroscopy platform, we explored these effects among a set of 143 hybrid indica rice varieties with different eating qualities. The single-grain amylose content (SGAC) and single-grain protein content (SGPC) models were established with coefficients of determination (R^2^) of 0.9064 and 0.8847, respectively, and the dispersion indicators (standard deviation, variance, extreme deviation, quartile deviation, and coefficient of variation) were proposed to analyze the variations in the SGAC and SGPC based on the predicted results. Our correlation analysis found that the higher the variation in the SGAC and SGPC, the lower the eating quality of the hybrid indica rice. Moreover, the addition of the dispersion indicators of the SGAC and SGPC improved the R^2^ of the eating quality model constructed using the composition-related physicochemical indicators (amylose content, protein content, alkali-spreading value, and gel consistency) from 0.657 to 0.850. Therefore, this new method proved to be useful for identifying high-eating-quality hybrid indica rice based on single near-infrared spectroscopy prior to processing and cooking.

## 1. Introduction

Rice (*Oryza sativa* L.) is one of the world’s most predominant food crops and is the food for approximately half of the world’s population, and it is divided into indica and japonica rice under long-term natural selection and artificial domestication; the indica rice has a higher amylose content (AC) and a slightly harder texture compared to the japonica rice. Eating quality is an important index with respect to consumers’ choice of rice variety, and it is also an essential factor determining its market value in addition to yield [1,2]. Hybrid rice has been cultivated in more than 300 countries and regions, including those in Asia, Africa, and Latin America, with an annual planting area of 22 million hectares and an average yield of 9 t/hm^2^, which is more than 20% higher than that of conventional rice [3]. Therefore, hybrid rice makes a significant contribution to world food security.

Eating quality is a comprehensive evaluation of the texture and flavor of cooked rice, and genetic and environmental factors have a strong influence on the eating quality [1,4,5]. Compared with conventional rice, the eating quality of hybrid rice is more complex because the hybrid rice is a segregated population of F_2_ due to the genetic differences in the parental lines. The AC, protein content (PC), alkali-spreading value (ASV), and gel consistency (GC) are composition-related physicochemical indicators of rice breeding and are highly correlated with eating quality. Among them, AC and PC are the main components of rice, and cooked rice with a high AC and PC is harder and less viscous, while that with low AC and PC is softer, more viscous, and better tasting; the ASV and GC are evaluations of the characteristics of starch and are positively correlated with rice’s eating quality [1,2,4,5]. However, the breeders and consumers have found that the eating quality of hybrid rice with similar physicochemical indicators varies substantially [6,7,8]. This may be because these indicators were obtained chemically from large population samples that were averaged over multiple single grains, and the composition of individual rice grains is inconsistent, which directly affects the eating quality of hybrid rice [9,10]. To verify this view, a high-throughput detection of rice composition at the single-grain level is needed. The traditional chemical detection technique for single-grain rice composition is destructive, expensive, inefficient, and insufficient to support the simultaneous detection of multiple components [11]. Therefore, the development of a high-throughput nondestructive detection technique for determining single-grain rice composition became the basis for conducting this study.

Near-infrared (NIR) spectroscopy is a rapid and nondestructive technique that can detect multiple chemical constituents of a sample and has been widely used in single-grain quality detection [12,13,14,15,16]. Models of single-grain amylose content (SGAC) and single-grain protein content (SGPC) were established successfully based on commercial NIR spectroscopy instruments, but the detection speed was slow [12,14]. Therefore, the NIR spectroscopy single-grain detection technique was feasible for the rapid and nondestructive prediction of the compositions of individual rice grains. Additionally, NIRS has been applied to detecting the eating quality of cooked rice. However, the studies on different rice varieties used milled rice or cooked rice and have not explored the variation among individual rice grains [15]. Alternatively, the study that explored the variation among individual rice grains focused on just one variety [16].

The main objective of this study was, based on a self-developed NIR spectroscopy single-grain detection platform, to determine the effects of variations in the chemical composition of individual rice grains on the eating quality of hybrid rice and to provide a new method for screening high-eating quality varieties in hybrid rice breeding. This will provide a theoretical basis for breeding high-quality rice. Since the hybrid indica rice is currently the most widely produced hybrid rice, the following studies were conducted to achieve these goals: (i) to develop the SGAC and SGPC models based a single-grain NIR spectroscopy platform to analyze the variations in the SGAC and SGPC of hybrid indica rice; (ii) to study the effects of the variations in single-grain composition on eating quality and establish an eating quality model, as well as the characteristics of the variations in the SGAC and SGPC with different eating qualities of hybrid indica rice. 

## 2. Materials and Methods

### 2.1. Materials

To make the NIR models more widely applicable, rice materials from different years were selected for modeling. Materials were provided by the breeding unit in Anhui Province from 2016 to 2020, of which 378 AC materials were used, with 70, 7, 108, 128, and 65 samples from 2016, 2018, 2019, 2020, and 2021, respectively. In total, 359 protein materials were used, with 236, 38, and 85 samples from 2016, 2019, and 2021, respectively.

A total of 148 hybrid indica rice varieties of different maturity stages—with 37 hybrid early-season rice samples and 111 hybrid middle-season rice samples—were used to analyze the correlation between the variation in the composition of individual grains and eating quality based on NIR models. The hybrid indica rice and parental lines were planted in the same experimental field in Hefei (31°52′ N, 117°17′ E) during 2020.

The NIR-modeling materials, hybrid indica rice, and parental lines were dried to a moisture of approximately 13% after harvest, then stored at 4 °C, and taken out to balance moisture for 2 weeks before the experiment.

### 2.2. Determination of Rice Physicochemical Indicators

The AC in milled rice was determined at 620 nm according to the iodine color development reaction [17]. Nitrogen content in brown rice was determined according to the Kjeldahl method, with Nitrogen content × 5.95 being the PC of the sample [9]. ASV and GC were measured according to the methods of Yang et al. [4]. Three replicates were performed for each measurement.

### 2.3. NIR Spectral Acquisition System of Individual Rice Grains

**Figure 1:**
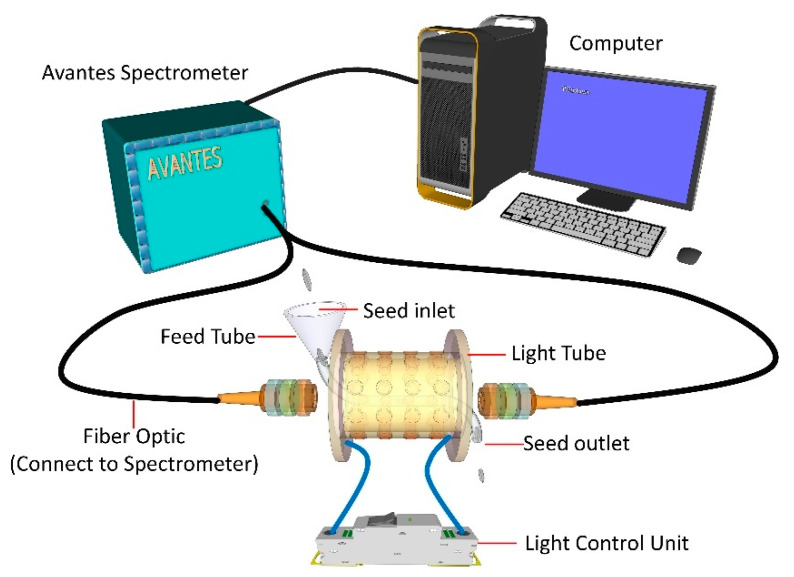
The high-throughput near-infrared spectral acquisition system of individual rice grains.

The diffuse reflectance spectra of individual rice grains were acquired using a high-throughput NIR spectral acquisition system developed in the laboratory (Figure 1); the acquisition system consisted of a spectrometer (Avantes, Apeldoorn, The Netherlands), computer, and a spectral collector providing a wavelength range of 1100–2500 nm with a resolution of 6.38 nm; and the original reflectance (R) was transformed into absorbance by inverse-log reflectance (log(1/R)). The spectral collector consisted of two optical fibers connected to a glass feed tube surrounding 64 tungsten halogen lamps with a collection speed of 2–3 grains per second. The spectral acquisition and analysis were measured according to Li et al. [18].

The self-developed parts of the instrument were the light tube and software. The light tube was applied for a Chinese patent, and the software was developed based on C++, which can control the self-designed circuit board to realize the control of the lights’ brightness and turning the lights on/off. The control and real-time acquisition of the spectrometer were realized by invoking the secondary development package of the spectrometer, and the acquisition, preprocessing, and calculation of the single-grain spectrum were achieved after setting a certain threshold value.

Ten rice grains with different grain types and contents were selected and scanned 10 times for each grain, and the model was used to predict the results of each repetition to analyze the population standard deviation of each model when predicting single grains [19].

### 2.4. Spectral Acquisition and Modeling Methods

The average spectra of multiple individual rice grains and the corresponding chemical values of the rice population can be used to establish a nondestructive NIR spectroscopy method for the composition analysis of individual rice grains [20]. The adopted method can reduce the error caused by too small of a weighing sample when directly detecting the components of single-grain rice. A total of 27 plump grains (with husks) were selected for each sample, and one NIR spectrum was collected for each grain. The spectra of 27 grains from each sample were averaged to produce the sample spectrum, and the samples were divided into calibration and validation sets based on the Kennard–Stone method to ensure a uniform distribution [21].

Different preprocessing methods spectral bands, and factors were selected to build different models using cross-tests in the calibration set, and the validation set was applied to evaluate the model’s performance. The pretreatment methods of constant offset elimination, vector normalization, first-order derivative (17-point smoothing), multiplicative scatter correction, and the combination operations with the first derivative were used to reduce the spectral variations caused by instrument noise, rice grain shape, and baseline drift.

The coefficient of determination of calibration (R^2^_cal_) and prediction (R^2^_p_), root mean square error of cross-validation (RMSECV) and prediction (RMSEP), relative percent deviation (RPD), and the average deviation between NIRS-predicted values and chemically determined values (bias) were used to predict the effectiveness of the generated model. Higher R^2^ and lower RMSE indicate a good model, while an RPD above 2.0 indicates stronger model reliability [13,15].

### 2.5. Analysis of the Variation in Composition of Individual Rice Grains

Considering the number of grains in a single spike of hybrid rice and the detection time, 300 rice grains were selected for each hybrid indica rice variety. The spectra were collected for each grain, and the AC and PC of each grain were predicted using the NIR models. The discrete analysis method was used to analyze the variation trend in the single-grain composition, and the dispersion indicators were standard deviation (SD), variance, range, quartile deviation (QD), and coefficient of variation (CV). Among them, the SD and variance indicate the degree of deviation and dispersion of all values in the dataset from the mean, respectively; QD is the value at 3/4 of the dataset subtracted from the value at 1/4 if the dataset is arranged in ascending order, which avoids the effect of abnormally large and small values; and CV can compare the degree of difference between datasets with different units of measurement or the same SD. The smaller the dispersion indicator, the lower the variation in SGAC and SGPC.

### 2.6. Detection Method of Eating Quality

The taste analyzer is an objective evaluation instrument that uses an internal NIR spectroscopy instrument in combination with sensory evaluation for calculations. The taste values of cooked rice from 148 hybrid indica rice varieties were measured using the Chinese indica model included in the Taste Meter (STA-1A Satake, Tokyo, Japan) with three replicates, and the taste value was then used to establish the eating quality model as a reference value [5,22]. The relationship between taste value and eating quality is defined by the following scale: ≤50 (very poor), 51–60 (poor), 61–70 (passing), 71–80 (relatively good), 81–90 (good), and >90 (very good).

Sensory evaluation is an accurate but subjective method for evaluating eating quality, and the sensory score was used to verify the accuracy of the eating quality model. The sensory score was evaluated according to “GB/T 15682–2008 sensory evaluation method for cooking and eating quality of milled rice” published by the Ministry of Agriculture of China [8,23]. Twenty-one professional tasters of different ages were selected to score the odor, appearance, structure, palatability (stickiness, elasticity, and softness), taste of cooked rice, and texture of cold cooked rice to obtain sensory scores. A conventional indica rice variety (Yuzhenxiang, with good eating quality and a taste value of 85.2) was used as a control in the sensory evaluation. 

### 2.7. Data Analysis

SPSS (IBM, Armonk, NY, USA) and Origin 2018 (Origin Lab, Northampton, MA, USA) were used for data statistics, correlation analysis, multiple regression analysis, and mapping, and OPUS 6.5 software (Bruker, Ettlingen, Germany) was used for spectral analysis and modeling.

## 3. Results

### 3.1. Chemical Composition Model of Individual Rice Grains

According to the KS algorithm, the samples for the SGAC and SGPC models were divided into two groups: 75% of the dataset made up the calibration set, and the other 25% formed the validation set. The descriptive statistics of the calibration and validation sets used in this study are shown in Table 1. The distribution of the calibration set was narrower than that of the validation set in both the SGAC and SGPC samples, which was suitable for model prediction.

**Figure 2:**
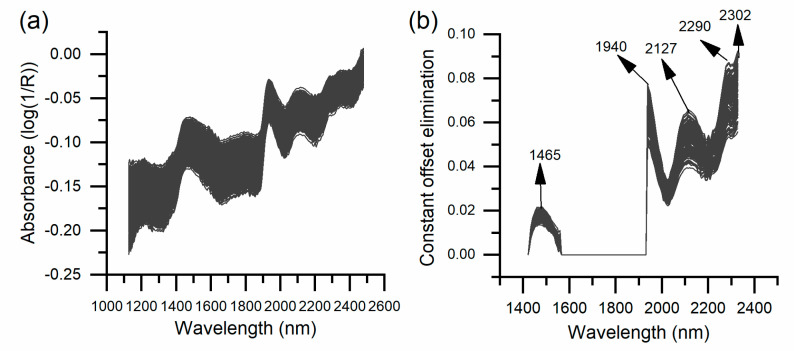
Near-infrared raw spectra (**a**) and vector normalized spectra (**b**) of individual rice grains.

The pretreatment methods were selected and combined with different wavelength ranges to model the SGAC and SGPC using the calibration set samples, and the modeling effect was determined by the prediction set (Table 2). For the partial least squares SGAC model, the full spectrum and without pretreatment produced a higher R^2^_cal_ and R^2^_p_ (0.9064 and 0.8628, respectively) and a lower RMSECV and RMSEP (1.54% and 1.7%, respectively). The full spectra of rice used in the SGAC model are shown in Figure 2a. Compared with other SGPC models, the pretreatment of constant offset elimination obtained a higher R^2^_cal_ and R^2^_p_ of 0.8847 and 0.8895, and two bands were chosen, which were located at 2327–1936.8 nm and 1559.3–1420.8 nm. A total of five feature peaks of 1465, 1940, 2127, 2290, and 2302 nm were found (Figure 2b).

### 3.2. Correlation Analysis of Single-Grain Chemical Composition Variations and Eating Quality of Hybrid Indica Rice

#### 3.2.1. The Taste Value, Physicochemical Indices, and Variation in the Single-Grain Chemical Composition of Hybrid Indica Rice 

**Figure 3:**
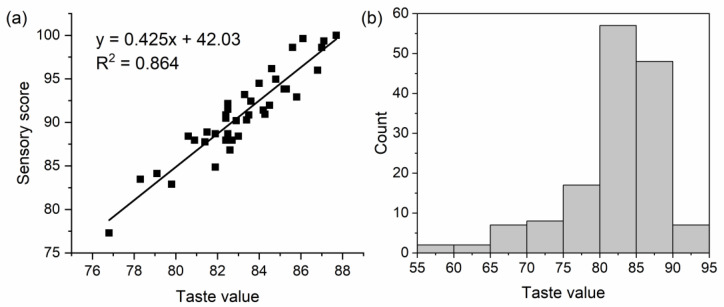
The regression analysis between taste values and sensory scores (**a**), and the distribution of taste value of 143 hybrid indica rice (**b**).

The regression analysis of the 39 hybrid indica rice varieties showed consistent overall trends between sensory evaluation and taste values, consistent with the high R^2^ of 0.864 (Figure 3a). Thus, the taste value can effectively evaluate the eating quality of hybrid indica rice. Among the 143 hybrid indica rice varieties, the distribution of their taste values is shown in Figure 3b. The range, mean, and CV of the taste values were 57.8–92.5, 82.2, and 7.8%, respectively, indicating that the eating quality of these hybrid rice varied widely. 

**Figure 4:**
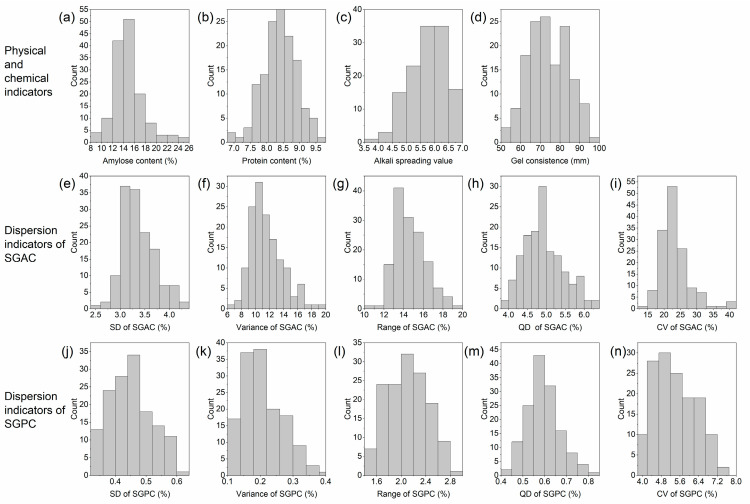
Distribution of physicochemical indices (amylose content, protein content, alkali-spreading value, and gel consistency) and dispersion indicators (SD, variance, range, quartile deviation (QD), and CV) of SGAC and SGPC of hybrid indica rice (*n* = 143). (**a**–**d**): physicochemical indicators, (**e**–**i**): dispersion indicators of SGAC, and (**j**–**n**): dispersion indicators of SGPC.

The distributions of the physicochemical indices and dispersion indices of the SGAC and SGPC of hybrid indica rice are shown in Figure 4. The ranges of the AC, PC, ASV, and GC were 9.0–24.9%, 6.9–9.8%, 3.9–7.0, and 43–96 mm, respectively, with CVs of 18.7%, 6.1%, 12.3%, and 14.4%, respectively, which indicated the large variation in AC among the physicochemical indicators of hybrid indica rice. In addition, the distribution of the SGAC and SGPC dispersion index values suggested a large difference between the different hybrid indica rice varieties (Figure 4e–n).

#### 3.2.2. Correlations between Taste Value and the Variation in SGAC and SGPC in Hybrid Indica Rice

The correlation between the physicochemical indicators, the dispersion indicators of SGAC and SGPC, and the taste value is shown in Table 3. The correlation analysis revealed that the physicochemical indicators were highly correlated with the taste value. Among the dispersion indicators of the SGAC, except for the CV, the others were negatively correlated with the taste value, and the CV was the opposite. None of the dispersion indicators of the SGPC were correlated with the taste value, apart from the QD, which had a significant negative correlation with the taste value. There was no correlation between the dispersion indicators of the SGPC and the taste value, except for the QD, which had a significantly negative relationship with that. Therefore, in addition to the physicochemical indices, the variations in the SGAC and SGPC were also associated with the eating quality of hybrid indica rice.

Due to the wide distribution of the AC and PC, the hybrid indica rice varieties with similar AC (11–14% and 14–17%) or PC (7.6–8.1%, 8.1–8.6%, and 8.6–9.1%) were selected, respectively, and the taste value was correlated with the corresponding indicators. From Table 4, it was determined that the SGAC dispersion indexes of the hybrid indica rice with similar AC were all negatively correlated with the taste value; when the PC was low, there was no correlation between the single-grain variation and taste value, and only when the PC was high and close (8.6–9.1%) were the dispersion indexes—in addition to the range of the SGPC—all significantly and negatively correlated with the taste value. 

#### 3.2.3. Effects of Taste Value and the Variation in SGAC and SGPC on Taste Value in Hybrid Indica Rice

**Figure 5:**
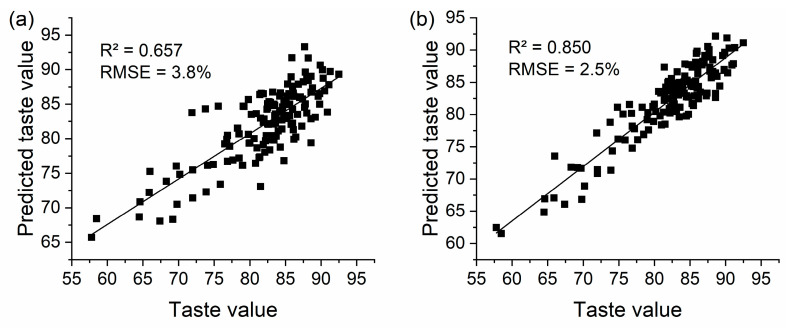
The eating quality model of hybrid indica rice using physicochemical indicators (**a**), and variations in SGAC and SGPC and physicochemical indicators (**b**).

Due to the strong correlation between the indicators associated with taste values (Table 3), the variables without collinearity were selected using a principal component analysis to evaluate the eating quality, and to determine the relative importance of the physicochemical indicators and the variations in the single-grain composition on eating quality of hybrid indica rice (Table S1). The R^2^ of the eating quality based on the physicochemical indicators was 65.7% (Figure 5a), and when the dispersion indicators in the SGAC and SGPC were added to the variables, the variance, range, and QD of SGPC, along with the variance of the SGAC, were chosen as independent variables. The R^2^ of the model increased to 85.0%, and the RMSE decreased from 3.8% to 2.5% (Figure 5b). 

The equation of the model was:

(1) TV=116.307−0.868×AC−1.886×PC+1.714×ASV+0.072×GC−1.338×SGACV+27.962×SGPCV−0.779×SGPCR−16.083×SGPCQ

where TV: Predicted taste value, AC: amylose content, PC: protein content, ASV: alkali-spreading value, GC: gel consistency, SGACV: Variance of SGAC, SGPCV: Variance of SGPC, SGPCR: Range of SGPC, and SGPCQ: Quartile deviation of SGPC.

#### 3.2.4. The Statistics of Composition-Related Indicators of Indica Hybrid Rice with Different Eating Qualities

**Figure 6:**
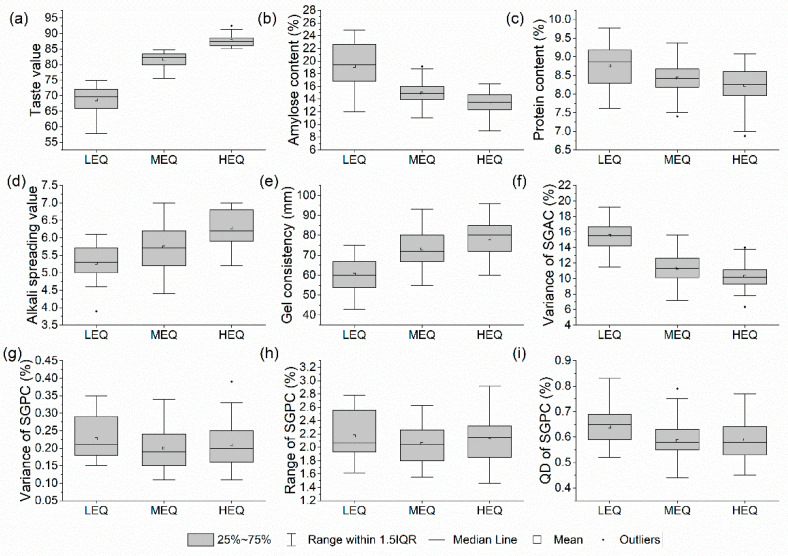
The box plots of taste value (**a**), physicochemical indicators (**b**–**e**), dispersion indices of SGAC and SGPC (**f**–**i**) for low eating quality (LEQ, taste value ≤ 75), medium eating quality (MEQ, 75 < taste value ≤ 85), and high-eating-quality (HEQ, taste value > 85) hybrid indica rice.

To facilitate the analysis of the related composition indicator-characteristics of the individual grains of hybrid indica rice with different eating qualities, the eating quality of hybrid indica rice was redefined into three categories based on the taste value of conventional indica rice Yuzhenxiang, which has a good sensory evaluation: below 75 (low eating quality), 75–85 (medium eating quality), and above 85 (high eating quality), and the sample sizes of the three eating quality categories were 19, 71, and 53, accounting for 13.3%, 49.7%, and 37.1%, respectively. The distribution characteristics of the physicochemical indicators and variations in the SGAC and SGPC related to the eating quality of each group are shown in Figure 6. We found that the AC, PC, variance of the SGAC, and the variance and QD of the SGPC decreased significantly with increasing taste values and were lower than 16.4%, 9.1%, 14.0%, 0.33%, and 0.77%, respectively, in the high-eating quality groups (Figure 6b,c,f,g,i). Meanwhile, the ASV and GC increased and were higher—in the high eating quality hybrid indica rice—than 5.2 and 60 mm, respectively (Figure 6d,e). However, the range of the SGPC was the widest, covering the lowest and highest values of all the samples.

## 4. Discussion

### 4.1. The Established NIR Models of SGAC and SGPC

The RPDs of the SGAC and SGPC models were higher than 2.5, indicating their stronger predictive ability [13,16].

The SGAC model with full spectra without processing was better than the other pretreatment models, which may be attributed to the wide source and variation of the modeled materials. For the SGAC of the rice grains, the results were superior to those of the AC model of milled rice built by Xia et al. [12] since the spectra they collected were for population milled rice of each sample, with gaps between each milled rice, and slightly inferior to those of the SGAC model of milled rice established by Siriphollakul et al. [16] because of the narrower spectral range, and the effect of the rice husk on the spectrum in the current research.

For the SGPC model, the peaks at 1465, 2127, 2290, and 2302 nm were protein-related absorption peaks, and the peak at 1940 nm was a water-related absorption peak, as proteins in the endosperm strongly interact with water [24,25,26]. The SGPC model’s results were similar to those based on the population rice grain spectrum using hyperspectral imaging [27], and slightly worse than the diffuse transmission SGPC model built by Xu et al. [11] because of the difference in the spectral acquisition methods, e.g., the diffuse reflectance spectra that were used in the paper.

The results presented here demonstrated that the SGAC and SGPC of rice grains could be determined accurately and nondestructively based on the self-developed throughput NIR spectroscopy platform.

### 4.2. Correlation Analysis of Single-Grain Chemical Composition Variations and Eating Quality of Hybrid Indica Rice

Based on chemical methods, Siriphollakul et al. [16] found that the polarity difference and SD of 154 grains of Khao Dawk Mali 105 were 14.48% and 2.68%, respectively, and Liu et al. [28] reported that the polarity differences in SGPC at different grain positions of different japonica rice varieties varied from 0.5% to 2.11%. These results indicated that differences in the single-grain compositions were similar to those using the chemical methods in this study. The population standard deviation of the predicted SGAC and SGPC (1.9% and 0.2%, respectively) from the platform were lower than the SD of single-grain hybrid indica rice (Table S2), indicating that the results of the variations in individual grains’ chemical composition were not caused by instrument or model deviation. Therefore, the prediction results of the NIR model for the SGAC and SGPC were reasonable and usable.

The correlations between the composition-related physicochemical indicators and taste values are consistent with previous studies, where a high AC and PC had a negative effect on the eating quality of cooked rice, and ASV and GC were positively correlated with eating quality [4,5]. Among the dispersion indicators of the SGAC, the taste value was positively correlated with the CV, while the others were negatively correlated, probably due to the wide distribution of AC (Table 3 and Figure 4). The analysis of samples with close AC or PC in hybrid indica rice supports this conclusion (Table 4); the lower the differences in the chemical composition of individual grains, the higher the eating quality when the composition was close. Furthermore, the correlation also showed that the SGAC had a stronger effect on eating quality than the SGPC and that the PC affected eating quality more strongly than the SGPC. This was because although the AC and PC in rice were influenced by both genetic and environmental factors, amylose was more heritable than protein and less influenced by the external environment than protein, and the variation among the SGPC values was unstable enough [29,30], which led to a stronger effect of the SGAC and PC on taste quality than the SGPC. In the regression analysis, only the variance was used to represent the variation trend of the SGAC, while the variance, range, and QD were selected to represent the variation trend of the SGPC to evaluate the eating quality, indirectly proving this view.

The R^2^ of the evaluation model of the rice’s eating quality—constructed based on physicochemical indicators and single-grain composition variation—in this study was 0.850, which is better than that based on the texture profile of cooked rice using near-infrared spectroscopy reported by Srisawas et al. [15] (R^2^ of 0.802), and Kim et al. [22] based on physicochemical indicators (R^2^ of 0.657) for evaluating rice’s eating quality. This may be because these studies were evaluated from a population perspective, while genetic and external environmental influences contributed to quality differences not only among the rice populations but also among individual grains. In particular, the edible hybrid rice originates from the seeds (F_2_) from F_1_ plants obtained via cross-fertilization, and the endosperm quality of F_2_ segregates due to the differences between parental lines; thus, the quality difference between single grains is more obvious [10].

The distribution of physicochemical indices of high-taste-quality hybrid indica rice was consistent with the “NY/T 593-2021 Cooking rice variety quality” published by the Ministry of Agriculture of China [31], and the distribution of variance of the SGAC and QD of SGPC provided the basis for the screening of high-eating-quality hybrid indica rice using the NIR spectroscopy single-grain platform.

## 5. Conclusions

In this study, based on single-grain NIR spectroscopy, we found that the compositional variation of individual rice grains was a new factor affecting the eating quality of hybrid rice. When the dispersion indicators of the SGAC and SGPC (the variance, range, and QD of SGPC, and the variance of the SGAC) were added to the eating quality model as independent variables, more accurate predictions could be achieved, with an increase in R^2^ of nearly 0.2 compared with using composition-related physicochemical indicators as independent variables. Since the single-grain NIR spectroscopy used in this study is fast and does not require the pretreatment of rice grains, it is more economical and rapid than the traditional detection method [8,9,17]. In addition, in contrast to NIR detection methods based on cooked rice or refined rice for the evaluation of taste quality [15,22], the method proposed in this study is a new method for evaluating rice taste that is more suitable for rapid detection and has complementary properties.

Due to the limited ranges of the physicochemical indicators and eating quality models used in this paper, and the fact that only hybrid indica rice was studied, this method can only be applied to the hybrid indica rice varieties within the ranges of the established models. Thus, we will supplement the modeling samples and study the hybrid japonica rice later to extend the application of this method, and because of the importance of single-grain components in breeding, we will develop a fully automated NIRS high-throughput quality sorter to achieve the screening of target traits.

## Figures and Tables

**Table 1:** Statistics of amylose content and protein content of NIR single-grain composition models.

		*n*	Mean	Min	Max	Standard Deviation (SD)	Coefficient of Variation (CV, %)
Calibration Set	Amylose content (%)	284	13.7	1.3	27.0	5.0	36.8
	Protein content (%)	269	8.6	6.4	10.3	0.8	9.3
Prediction Set	Amylose content (%)	94	14.2	1.7	23.6	4.6	32.5
	Protein content (%)	90	8.5	6.9	9.8	0.8	9.6

**Table 2:** Analysis of partial least squares models of rice single-grain amylose content (SGAC) and single-grain protein content (SGPC) using different pretreatments and spectral ranges.

SGAC (%)									
Pretreatment		None	None	COE ^a^	1 der ^b^	SNV ^c^	MSC ^d^	1 der + SNV	1 der + MSC
Factors		13	12	12	13	9	10	10	11
Calibration set	R^2^_cal_	0.9064	0.8528	0.8414	0.835	0.7829	0.7915	0.7692	0.7638
	RMSECV	1.54	1.93	2	2.04	2.34	2.29	2.41	2.44
	RPD	3.27	2.61	2.51	2.46	2.15	2.19	2.08	2.06
	bias	−0.00532	−0.0239	−0.0209	−0.0136	−0.0072	−0.01	−0.00853	0.00979
Prediction set	R^2^_p_	0.8628	0.8537	0.8429	0.7762	0.8298	0.8309	0.7549	0.7614
	RMSEP	1.7	1.75	1.82	2.17	1.89	1.88	2.27	2.24
	RPD	2.79	2.68	2.54	2.12	2.47	2.45	2.03	2.07
	bias	−0.438	−0.398	−0.232	−0.215	−0.348	−0.195	−0.222	−0.311
Wavelength ranges (nm)		2500–1100	2452.8–1188	2327–2194.8, 2075.3–1188	2452.8–1427.1, 1307.6–1175.4	2075.3–1685.1, 1559.3–1175.4	2327–1188	2327–2069, 1949.4–1691.4, 1571.9–1188	2327–2194.8, 1949.4–1188
**SGPC (%)**									
**Pretreatment**		**None**	**None**	**COE ^a^**	**1 der ^b^**	**SNV ^c^**	**MSC ^d^**	**1 der + SNV**	**1 der + MSC**
Factors		6	12	12	13	8	9	12	10
Calibration set	R^2^_cal_	0.8321	0.8564	0.8847	0.8409	0.8351	0.8483	0.8308	0.8182
	RMSECV	0.329	0.304	0.273	0.32	0.326	0.313	0.33	0.342
	RPD	2.44	2.64	2.94	2.51	2.46	2.57	2.43	2.35
	bias	0.00037	0.00196	0.00168	0.00177	0.0021	0.00666	0.00244	0.00227
Prediction set	R^2^_p_	0.8084	0.8496	0.8895	0.838	0.8412	0.8383	0.8217	0.8323
	RMSEP	0.378	0.335	0.287	0.348	0.344	0.347	0.365	0.354
	RPD	2.29	2.58	3.01	2.48	2.51	2.49	2.37	2.44
	bias	−0.0146	0.0149	−0.0169	0.00433	0.00405	0.0185	0.0125	−0.00753
Wavelength ranges (nm)		2500–1100	2201.1–1553, 1301.3–1169.1	2327–1936.8, 1559.3–1420.8	2201.1–2062.7, 1943.1–1420.8, 1301.3–1169.1	2327–2062.7, 1811–1553	2452.8–2062.7, 1685.1–1553	2452.8–2320.7, 2201.1–2062.7, 1559.3–1420.8	2327–2062.7, 1811–1553

^a^ Constant offset elimination, ^b^ First derivative, ^c^ Vector normalization, and ^d^ Multiplicative scatter correction.

**Table 3:** Correlation coefficients between physicochemical indicators (amylose content (AC), protein content (PC), alkali-spreading value (ASV), and gel consistency (GC)), variation indicators (SD, variance, range, QD, and CV) in single-grain chemical composition, and taste value of hybrid indica rice.

		Taste Value	Physicochemical Indicators				SGAC					SGPC				
			AC	PC	ASV	GC	SD	Variance	Range	QD	CV	SD	Variance	Range	QD	CV
Taste Value		1	−0.670 **	−0.376 **	0.525 **	0.562 **	−0.724 **	−0.744 **	−0.658 **	−0.660 **	0.222 **	−0.050	−0.045	0.034	−0.205 *	0.100
Physicochemical Indicators	AC		1	0.153	−0.241 **	−0.448 **	0.346 **	0.361 **	0.303 **	0.354 **	−0.772 **	−0.022	−0.030	−0.026	−0.004	−0.082
	PC			1	−0.268 **	−0.283 **	0.170 *	0.188 *	0.169 *	0.148	−0.06	−0.010	−0.014	−0.033	−0.024	−0.382 **
	ASV				1	0.316 **	−0.335 **	−0.342 **	−0.259 **	−0.385 **	0.039	−0.029	−0.025	0.040	−0.083	0.080
	GC					1	−0.325 **	−0.342 **	−0.255 **	−0.289 **	0.238 **	−0.069	−0.068	−0.003	−0.172 *	0.048
SGAC	SD						1	0.998 **	0.896 **	0.900 **	0.273 **	0.288 **	0.287 **	0.147	0.372 **	0.196 *
	Variance							1	0.901 **	0.898 **	0.258 **	0.282 **	0.281 **	0.147	0.369 **	0.184 *
	Range								1	0.760 **	0.259 **	0.273 **	0.273 **	0.220 **	0.340 **	0.185 *
	QD									1	0.204*	0.261 **	0.262 **	0.142	0.339 **	0.181 *
	CV										1	0.182 *	0.195 *	0.124	0.203 *	0.191 *
SGPC	SD											1	0.997 **	0.893 **	0.788 **	0.926 **
	Variance												1	0.893 **	0.787 **	0.924 **
	Range													1	0.642 **	0.833 **
	QD														1	0.737 **
	CV															1

* and ** denote significant differences at the 0.05 and 0.01 levels, respectively.

**Table 4:** Correlation coefficients between physicochemical indicators, variation indicators (SD, variance, range, QD, and CV) in SGAC and SGPC, and taste value of hybrid indica rice with similar composition.

	Physicochemical Indicators				SGAC Dispersion Indicators					SGPC Dispersion Indicators				
	AC	PC	ASV	GC	SD	Variance	Range	QD	CV	SD	Variance	Range	QD	CV
AC of 11–14% (*n* = 50)	0.032	−0.165	0.507 **	0.136	−0.646 **	−0.665 **	−0.591 **	−0.585 **	−0.476 **	−0.001	−0.044	0.071	−0.230	0.056
AC of 14–17% (*n* = 67)	−0.059	−0.465 **	0.470 **	0.462 **	−0.490 **	−0.502 **	−0.399 **	−0.335 **	−0.328 **	−0.048	−0.027	−0.009	−0.158	0.129
PC of 7.6–8.1% (*n* = 28)	−0.727 **	0.292	0.608 **	0.458 *	−0.282	−0.283	−0.198	−0.262	0.439 *	0.312	0.306	0.284	0.123	0.260
PC of 8.1–8.6% (*n* = 62)	−0.595 **	−0.045	0.366 **	0.424 **	−0.686 **	−0.706 **	−0.650 **	−0.568 **	0.142	0.024	−0.006	0.093	−0.078	0.024
PC of 8.6–9.1% (*n* = 35)	−0.777 **	−0.024	0.540 **	0.672 **	−0.821 **	−0.832 **	−0.704 **	−0.793 **	0.235	−0.425 *	−0.436 **	−0.307	−0.680 **	−0.416 *

* and ** denote significant differences at the 0.05 and 0.01 levels, respectively.

## Data Availability

The data that support the findings of this study are available from the corresponding author upon reasonable request.

## Supplementary Materials

The following supporting information can be found at https://www.mdpi.com/article/10.3390/foods11172634/s1. Supplementary Table S1. Summary of eating quality regression models of hybrid indica rice using physicochemical indicators^1^, and variations in single-grain composition and physicochemical indicators^2^. Table S2. The population standard deviation of the SGAC and SGPC models based on NIR spectroscopy platform.
