# Supplementary material for: Effects of Variations in the Chemical Composition of Individual Rice Grains on the Eating Quality of Hybrid Indica Rice Based on Near-Infrared Spectroscopy
Source: Foods. 2022 Aug 30;11(17):2634. doi: 10.3390/foods11172634 (PMC9455687; doi:10.3390/foods11172634)
Supplement: Supplementary file 1 [file foods-11-02634-s001.zip › Supplementary.pdf]

Table S1. Summary of eating quality regression models of hybrid indica rice using physicochemical indicators[1], and variations in single-grain composition and physicochemical indicators[2].

**1**	**Unstandardized coefficients**		**R^2**	**VIF of collinearity**	**2**	**Unstandardized coefficients**		**R^2**	**VIF of collinearity**
	B	Standard error				B	Standard error		
**(Constant)**	91.088	8.146	**0.657**		(Constant)	116.307	6.621	0.85	
**AC**	−1.126	0.132		1.269	AC	−0.868	0.093		1.407
**PC**	−2.095	0.678		1.131	PC	−1.886	0.460		1.151
**ASV**	2.735	0.491		1.170	ASV	1.714	0.340		1.243
**GC**	0.127	0.036		1.380	GC	0.072	0.025		1.463
					Variance of SGAC	−1.338	0.116		1.555
					Variance of SGPC	27.962	10.069		8.244
					Range of SGPC	−0.779	1.630		5.463
					QD of SGPC	−16.083	5.080		2.985

Dependent variable: taste value

Table S2. The population standard deviation of the SGAC and SGPC models based on the NIRS single-grain platform.

Sample NO.	1	2	3	4	5	6	7	8	9	10
Predicted SGAC for 10 repetitions (%)	10.37	10.79	5.89	2.89	21.32	14.32	7.65	22.45	10.56	17.95
	9.73	7.31	5.79	−1.52	20.10	15.05	4.46	22.48	13.93	16.74
	13.27	9.72	5.60	2.81	19.97	14.73	7.35	19.35	13.94	14.99
	13.12	8.51	9.58	−0.49	20.88	14.76	5.00	20.02	11.20	13.25
	14.16	9.77	6.34	2.44	22.86	14.78	0.09	19.74	11.03	15.68
	11.41	12.96	6.13	−0.72	20.08	10.55	2.28	20.01	19.41	14.86
	12.35	11.33	6.45	−0.75	22.45	14.25	3.45	20.61	11.95	16.77
	16.14	7.17	6.88	0.24	19.21	13.05	4.49	20.95	12.12	11.60
	14.11	7.33	7.27	−2.31	22.47	13.37	4.79	21.48	12.19	17.12
	13.76	8.43	6.98	2.30	23.92	12.82	1.70	19.31	12.32	17.25
SD (%)	1.93	1.94	1.15	1.95	1.53	1.38	2.36	1.18	2.55	1.99
Population standard deviation (%)	**1.9**									
Predicted SGPC for 10 repetitions (%)	8.00	7.63	7.76	8.08	9.05	7.91	7.98	9.06	9.23	7.09
	7.73	7.75	7.97	7.94	9.22	8.28	7.64	9.11	9.19	6.92
	7.88	7.90	7.75	8.01	9.18	7.89	7.76	8.94	9.67	7.21
	7.49	8.08	8.03	7.91	9.27	8.34	7.80	8.60	9.47	6.92
	7.61	7.79	7.91	7.94	9.41	8.66	8.09	9.24	9.69	7.09
	7.68	7.56	7.94	8.28	9.11	8.38	8.45	8.86	9.02	6.91
	7.68	7.96	7.96	8.40	9.41	8.13	7.77	8.75	9.37	6.65
	7.43	7.99	7.57	8.25	9.50	7.79	8.15	8.81	9.22	7.03
	8.03	7.62	7.72	8.13	8.98	8.02	7.61	9.09	9.10	7.14
	8.03	7.96	7.59	8.05	9.36	8.29	8.17	8.62	9.26	6.85
SD (%)	0.22	0.18	0.17	0.17	0.17	0.27	0.27	0.22	0.23	0.16
Population standard deviation (%)	**0.2**									
